# A Fatal Case of Disseminated Tuberculosis Coincident with Measles-Rubella Vaccination

**DOI:** 10.4178/epih/e2010002

**Published:** 2010-04-26

**Authors:** Hae-Kwan Cheong, Byung-Guk Yang, Young Mo Sohn, Il-Hoon Kwon, Jun Chul Kim, Hangmi Kim, Jung Ran Kim

**Affiliations:** 1 Department of Social and Preventive Medicine, Sungkyunkwan University School of Medicine, Suwon, Korea.; 2 Center for Communicable Disease Surveillance and Response, Korea Centers for Disease Control and Prevention, Seoul, Korea.; 3 Department of Pediatrics, Yonsei University College of Medicine, Seoul, Korea.; 4 National Institute of Scientific Investigation, Daejeon, Korea.; 5 Public Health, Social Welfare, and Women's Affairs Department, Daegu, Korea.; 6 Department of Pathology, Kyungpook University School of Medicine, Daegu, Korea.; 7 Department of Pathology, Dongguk University College of Medicine, Gyeongju, Korea.

**Keywords:** Measles, Vaccination, Tuberculosis, Adverse effects

## Abstract

The authors report a fatal case of disseminated tuberculosis in a 14-yr-old girl, which developed immediately after a measles-rubella (MR) vaccination. Despite a markedly accelerated clinical course which led to death within two weeks, the authors could not identify any possible cause of the tuberculosis aggravation in this case, with the exception of the MR vaccination. The possible role that MR vaccination had on the clinical course of tuberculosis in this case is discussed.

## INTRODUCTION

Natural measles infection can aggravate other infectious process, e.g., tuberculosis, because of its marked deteriorating effect on the immune system. However, it is unknown whether measles or rubella vaccination can activate tuberculosis infections, and no definite cases have been reported supporting this relationship. The recent reemergence and persistence of tuberculosis in developing countries suggests that this issue is of some importance to both clinical and public health practices.

Korea witnessed an explosive increase in the incidence of measles between March 2000 and June 2001, which culminated in more than 50,000 cases, mostly among children and adolescents [1, 2]. During the course of the mass measles-rubella (MR) catch-up vaccination, the authors experienced a fatal case of disseminated military tuberculosis in an adolescent girl after a MR vaccination, which involved the rapid progression of disseminated tuberculosis. Moreover, disease onset coincided with MR vaccination in this case.

## CASE PRESENTATION

**Figure 1:**
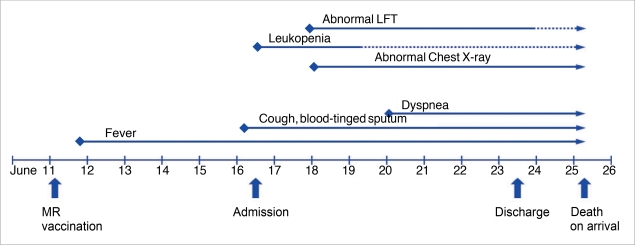
Flow chart of the clinical course and main events of the case. Main events described below the date line and clinical manifestations and laboratory findings are described above the date line.

**Figure 2:**
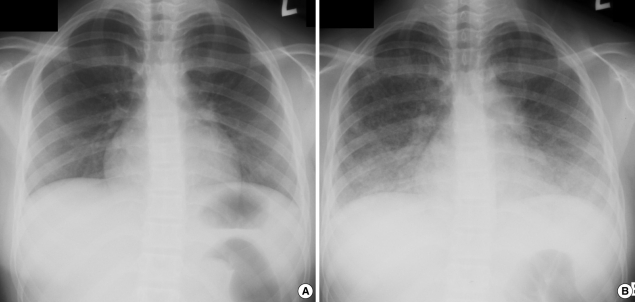
Chest X-ray findings. (**A**) Mild reticular densities were found in both lung fields (June 16th, 2006), and (**B**) increased reticular densities were mainly observed in both lower lung fields. Small nodular densities were newly developed, but neither cardiomegaly nor pleural exudates were observed. Perihilar lymph node enlargement was suspected (June 25th, 2006, postmortem).

A 14-yr-old girl developed a mild fever, a chilling sensation, and generalized myalgia from 9:00 pm on June 11th, 2001, after an MR vaccination on the afternoon of the same day (Figure 1). The next day, she developed a high fever of up to 39℃ accompanied by a chilling sensation. She visited a public health center and a clinic, took some cold medicine including antipyretics, but the fever persisted. She had a headache and nausea, which progressively aggravated in spite of medication. On June 16th, she was admitted to a hospital. On admission, she looked acutely ill and complained of headache and discomfort in the upper abdomen. Her body temperature was 39.1℃, and her pharynx was injected, but breathing sounds were normal, and there were no abnormal findings in abdomen. Her hemoglobin was 11.9 g/dL, white blood cell count 1,300/µL, platelet count 100,000/µL, and erythrocyte sedimentation rate (ESR) 10 mm/hr. Her urine contained many red blood cells due to menstruation. On the day of admission, she developed a cough with blood-tinged sputum. Chest and abdominal X-rays were normal (Figure 2A). After admission, despite antibiotic medication including penicillin and tetracycline derivatives, her fever was uncontrolled. Chest X-ray on the third day of admission (June 18th) showed pulmonary infiltration. Laboratory tests showed; aspartate aminotransferase (AST) 364 IU/L, alanine aminotransferase (ALT) 317 IU/L, alkaline phosphatase (ALP) 295 IU/L, gamma-glutamyl transpeptidase (GGT) 156 IU/L, with a normal bilirubin level (June 18th). Viral markers for hepatitis A, B, and C virus were all negative. The Widal test was 1:80 for O antigen and 1:160 for H antigen, negative for active infection. Blood smear for plasmodium was negative. A blood culture on the June 18th was negative. On the sixth day of admission (June 21st), she became slightly dyspneic and febrile, the headache, chill and cough persisted, but chest X-ray findings were unchanged. Sputum smear for acid-fast bacilli was negative with many Gram positive bacteria, and her liver enzyme levels were improved (AST 151 IU/L, ALT 157 IU/L). On the eighth day (June 23rd), because of her parent's strong insist on discharge, she was discharged against recommendation. Medications including oral antibiotics were prescribed. However, no antituberculous medication was given at any time during the clinical course.

On June 25th, two days after discharge, she was brought to the emergency department of a general hospital due to cardiorespiratory arrest. On arrival, she was unconscious, totally apneic, and generally cyanotic without any vital signs. On chest X-ray, both lungs were totally hazy (Figure 2B). On the following day, an autopsy was performed at the request of governmental health authorities.

She had contracted measles at 2 yr of age, and had not been vaccinated on measles prior to this vaccination; other vaccinations had been performed as scheduled. She had no history of tuberculosis or any other chronic illnesses.

On autopsy, she was well developed, 160 cm tall and with body weight of 63 kg, and a BCG scar was found on her left shoulder. The both lungs showed multiple randomly scattered foci of small yellow lesions with lobular and patchy pulmonary consolidation. Microscopically, miliary granulomas throughout the lungs were evident. The most of granulomas were noncaseating but some of these have central caseous necrosis. The granulomas consisted of epithelioid histiocytes, Langerhans giant cells and a few lymphocytes. Acid fast bacilli (AFB) stain was not done. The pathologic findings were compatible with tubeerculous infection. More peripherally of granulomas, hyaline fibrin membranous lining alveolar spaces and edema were present. These distinctive granulomas were also found in hilar lymph nodes, spleen and kidney.

Specific IgM antibody for measles was negative in serum samples on June 19th and 26th. However, IgG for measles was positive by enzyme-linked immunosorbent assay (ELISA) (3,000-3,400 IU/mL), but not elevated in the paired serum samples. Other antibodies for common viruses, including herpes simplex virus (IgM ELISA), cytomegalovirus (IgM ELISA), varicella zoster virus (IgM ELISA), and human immunodeficiency virus (Western blot) were negative for serum samples taken on the day of death. However, the CD4/CD8 ratio and mitogen-induced lymphocyte proliferation were decreased.

Viral antigen polymerase chain reaction (PCR) tests for measles virus, adenovirus, respiratory syncytial virus, parainfluenza virus, influenza virus, *Mycoplasma pneumoniae*, and enteroviruses were all negative for postmortem bronchial aspirate, blood, cerebrospinal fluid, and pulmonary fluid. However, PCR for acid-fast bacillus was positive for postmortem bronchial aspirate, lung, liver, brain, and peribronchial lymph nodes, but negative for cerebrospinal fluid and pericardial fluid. The AFB smear test was positive for postmortem bronchial aspirate, lung, liver, peribronchial lymph nodes, but negative for cerebrospinal fluid, brain, and pericardial fluid.

## DISCUSSION

The authors report a fatal case of disseminated tuberculosis that developed immediately after an MR booster vaccination.

During the diagnostic work up of this case, viral or mycoplasma pneumonia was excluded by viral antibody and microbiologic test findings. Viral hepatitis A, B, and C, which are prevalent in the Korean population, were also excluded based on the absence of viral markers. Autopsy findings confirmed the involvement of tuberculosis in multiple organ systems.

The infection could be differentiated from measles, because the patient's clinical features and the disease course were not compatible with measles infection. In addition to a history of measles infection at age two, a serologic examination failed to show an elevated IgM anti-measles level or an increase of IgG antibody level in paired sera, which is compatible with a history of measles infection in early childhood. Moreover, regional surveillance data showed no measles outbreak where she lived, although a nationwide measles outbreak did occur during that year 2001, and her parents and teachers denied any recent history of travel outside the community. Therefore, it is least possible that she could have contracted a natural measles infection before or at the same time as the tuberculosis infection.

The origin of the tuberculosis infection in this case was not evident immediately after death. Chest X-ray examinations of family members and students in her class failed to reveal any positive cases. However, two months after her death, her mother visited a hospital due to an intermittent cough, and although her chest X-ray was normal, a bronchoscopic examination has shown that she had bronchial tuberculosis without any previous history of tuberculosis. The mother had a BCG scar on her shoulder and her previous chest X-ray one year before her daughter's death was normal. These findings suggest that the tuberculosis might have been originated from the mother while she was asymptomatic or vice versa.

It has been reported that it takes two to six months for a primary infection of tuberculosis to progress to the disseminated form, and three to five weeks after hematologic dissemination for miliary nodules to be evident by chest X-ray [4]. In the present case, based on clinical symptom, onset of tuberculosis is estimated to be June 11th, and the first chest x-ray findings were evident in seven days after the development of clinical illness. Thus, in view of the natural course of disseminated tuberculosis, this case followed a highly accelerated course, i.e., death due to multiple organ involvement and an abrupt disease onset and detection occurred only in 13 days period.

In view of the presence of a BCG scar, such an extraordinary disease progression is likely to be associated with an immune compromised state. During the investigation of this case, however, there was no evidence of a predisposition to immune suppression, i.e., no immunosuppressive drugs had been administered, and there was no evidence of coexistent viral or bacterial infection, malignancy, or trauma, although she was in menstruation during admission. Moreover, the patient was well developed for her age, which excluded the possibility of malnutrition, and her immunoglobulin profile was within the normal range, which excluded the possibility of a humoral immunity disturbance. Human immunodeficiency virus (HIV) antibody was also negative.

In the course of natural measles infection, the potentiation of other infections, including tuberculosis, is common [5]. CD4 Th2 cells are mainly activated during natural measles infections [6], and increased secretion of IL-10 (a cytokine released by Th2 cells) suppresses DTH response, which induces a temporary anergy response to the tuberculin skin test [7]. Moreover, a decreased CD4/CD8 ratio, mitogen-induced lymphocytic proliferation and macrophage inactivation facilitate host susceptibility to infections [6].

A few reports have addressed tuberculosis activation after MR vaccination [8]. However, even among these cases, the actual potentiation of tuberculosis infection related to MR vaccination has been reported only in severely immune compromised subjects [9]. Reports on immune response modification in association with MR vaccination are very rare. High dose measles vaccinations in Africa during the 1990s were found to be associated with higher mortality in infants [10], due to infectious diseases, a reduced CD4/CD8 ratio, and mitogeninduced lymphocyte proliferation [10]. However, to the best of the authors' knowledge, no report has addressed the activation of tuberculosis by MR vaccination. Moreover, the vaccination guidelines of the World Health Organization [11], the American Pediatric Society [12], the Korean Association of Pediatrics, and the Korean Center for Disease Control and Prevention [13] do not recommend that a tuberculin test be conducted before MR vaccination. Despite a remarkable decrease in the severity and prevalence of tuberculosis, it was the 10th most common cause of death in the Korean population until 2004 [3]. However, physicians are less aware of tuberculosis during the differential diagnosis of infectious diseases. Notably, in the described case, a failure to diagnose tuberculosis resulted in a fatal outcome.

There is no definitive evidence that MR vaccination can activate tuberculosis, although the possibility cannot be denied. In this case, clinical symptoms developed immediately after vaccination. Considering the incubation period of tuberculosis, it is highly unlikely that MR catch-up immunization directly induced the tuberculosis infection, and thus it is more reasonable to presume that MR vaccination was done during the active stage of tuberculosis infection. However, the possibility that coincident MR immunization during the tuberculosis incubation period might have accelerated the clinical course is suggested by the protracted nature of the clinical course. Some of the host factors including debility, old age, chemotherapeutics, malignancy, corticosteroid therapy, local injury, and coincidental infection could predispose the rapid aggravation of the clinical course of the tuberculosis. The authors, however, were not able to find any factor other than MR vaccination that might have accelerated the clinical course in this case. Further monitoring is required to establish the nature of the relationship between MR vaccination and tuberculosis.
